# A systematic review of the incidence of hypersensitivity reactions and post-contrast acute kidney injury after ioversol in more than 57,000 patients: part 1—intravenous administration

**DOI:** 10.1007/s00330-022-08636-3

**Published:** 2022-03-21

**Authors:** Aart J. van der Molen, Ilona A. Dekkers, Ibrahim Bedioune, Elisabeth Darmon-Kern

**Affiliations:** 1 Contrast Media Safety Research Group, Department of Radiology C-2S, Leiden University Medical Center, Albinusdreef 2, NL-2333 ZA Leiden, The Netherlands; 2 Clinical Development Department, Guerbet, Roissy CDG Cedex, France

**Keywords:** Ioversol, Contrast media, Administration, intravenous, Acute kidney injury, Drug-related side effects and adverse reactions

## Abstract

**Objectives:**

To evaluate the incidence of adverse drug reactions (ADRs), including hypersensitivity reactions (HSRs) and post-contrast acute kidney injury (PC-AKI), after intravenous (IV) administration of ioversol.

**Materials and methods:**

A systematic literature search (1980–2021) of studies documenting IV use of ioversol and presence or absence of ADRs, HSRs, or PC-AKI was performed. Key information including patients’ characteristics, indication and dose of ioversol, safety outcome incidence, intensity and seriousness were extracted.

**Results:**

Thirty-one studies (> 57,000 patients) were selected, including 4 pediatric studies. The incidence of ADRs in adults was reported in 12 studies from ioversol clinical development with a median (range) of 1.65% (0–33.3%), and 3 other studies with an incidence between 0.13 and 0.28%. The incidence of HSRs (reported in 2 studies) ranged from 0.20 to 0.66%, and acute events (4 studies) from 0.23 to 1.80%. Severe reactions were rare with a median (range) of 0 (0–4%), and none were reported among pediatric patients. The incidence of ADRs and HSRs with ioversol, especially those of severe intensity, was among the lowest in studies comparing different iodinated contrast media (ICM) of the same class. PC-AKI incidence was variable (1–42% in 5 studies); however, ioversol exposure *per se* did not increase the incidence.

**Conclusions:**

When administered by the IV route, ioversol has a good safety profile comparable to that of other ICM within the same class, with a low incidence of severe/serious ADRs overall, and particularly HSRs. PC-AKI incidence does not seem to be increased compared to patients who did not receive ioversol. Further well-designed studies are warranted to confirm these results.

**Key Points:**

*• Ioversol has a good safety profile in adult and pediatric patients when IV administered.*

*• ADR and HSR incidence with ioversol, especially those of severe intensity, was among the lowest compared to other ICM.*

*• IV administration of ioversol per se did not increase PC-AKI incidence.*

**Supplementary Information:**

The online version contains supplementary material available at 10.1007/s00330-022-08636-3.

## Introduction

Iodine-based contrast media (ICM) are widely used in clinical practice for various X-ray-based modalities, and can be classified, according to their osmolality, into hyperosmolar CM (HOCM), low-osmolar CM (LOCM), and iso-osmolar CM (IOCM) [1]. They can be further subdivided into ionic and non-ionic CM, which do not dissociate into ions in water and are therefore lower in osmolality [2].

Ioversol (Optiray®, Guerbet) is a non-ionic, monomeric LOCM, with an osmolality between 502 and 792 mOsm/kg, depending on iodine concentration (240, 300, 320, or 350 mg I/mL).

Despite the generally good safety profile of ICM, adverse drug reactions (ADRs) may occur and can be life threatening. Among these reactions, there are hypersensitivity reactions (HSRs) [3]. Immediate (acute) HSRs occur within 1 h after ICM administration and may include urticaria, angioedema, bronchospasm, laryngeal edema, and anaphylactic shock. Non-immediate (delayed) HSRs, with symptoms occurring between 1 h and several days after ICM administration, commonly manifest as delayed urticaria and maculopapular exanthema, and rarely as severe cutaneous adverse reactions (SCARs) [3].

Post-contrast acute kidney injury (PC-AKI) is a complication that might occur after intravascular exposure to ICM. PC-AKI has been associated with excess morbidity and mortality [4–6], and chronic kidney disease (CKD) is the most well-known risk factor [7]. The risk of PC-AKI could increase from 5% at an estimated glomerular filtration rate (eGFR) ≥ 60 to 30% at an eGFR < 30 mL/min/1.73 m^2^ [8]. Several definitions of PC-AKI, based on serum creatinine (SCr) concentration, have been proposed by different initiatives, the European Society of Urogenital Radiology (ESUR) [9], the Acute Kidney Injury Network (AKIN) [10], and the Kidney Disease Improving Global Outcomes (KDIGO) being the most recent [11].

As the causal relationship between ICM exposure and the occurrence of AKI is often confounded by several patient- and procedure-related factors, the term PC-AKI is preferred for AKI associated with CM administration for studies lacking a control population [9]. Only when the ICM is demonstrated as the causative factor is the term contrast-induced acute kidney injury (CI-AKI) or contrast-induced nephropathy (CIN) appropriate.

To support radiologists in their clinical practice, we sought to perform this systematic analysis of literature on the incidence of ADRs, HSRs, and PC-AKI after intravenous (IV) administration of ioversol and to position the safety profile of ioversol among the different ICM. Complications after intra-arterial administration will be discussed in a future review.

## Materials and methods

This systematic literature review was performed according to the Preferred Reporting Items for Systematic Reviews and Meta-analyses (PRISMA) guidelines [12].

### Data sources and searches

A search of MEDLINE (PubMed) and EMBASE (Elsevier) references from January 1980 to May 2021 was performed using keywords related to adverse events usually associated with the use of ICM such as “allergic reaction,” “hypersensitivity,” “anaphylactic,” “nephrotoxicity,” and “kidney injury” (Appendix 1).

### Study selection

Clinical studies documenting exposure to IV ioversol and the presence or absence of ADRs, and/or HSRs, and/or PC-AKI were included. Systematic or descriptive reviews, commentaries, letters, or case reports were excluded. Studies with less than 5 patients exposed to ioversol were excluded.

Study selection was conducted and reconciled between two independent authors. After a first screening step of all identified references, based on titles and abstracts, a full-text screening of potentially relevant publications was performed. Additional relevant publications were identified by cross-referencing.

### Data extraction and study quality assessment

Key data extracted from selected articles were as follows: study design, patient characteristics, indication for which ioversol was used, number of patients exposed to ioversol and other ICM (if any) or number of administered doses, ICM dose, type of safety outcome and incidence, intensity [13] and seriousness if reported, and definition of PC-AKI (when applicable).

The methodological quality of the non-randomized studies was assessed using a modified Newcastle-Ottawa Scale (NOS) [14]. The score ranged from 0 to 8, based on 8 questions (one question excluded as not appropriate for safety outcomes) related to patient selection, comparability of cohorts, and outcomes assessment. Scores of 7–8 and 5–6 indicated high-quality and moderate-quality studies, respectively. The revised Cochrane Risk of Bias assessment tool for randomized trials (ROB 2) algorithm was used for randomized controlled trials (RCT) [15].

## Results

### Study selection

**Fig. 1:**
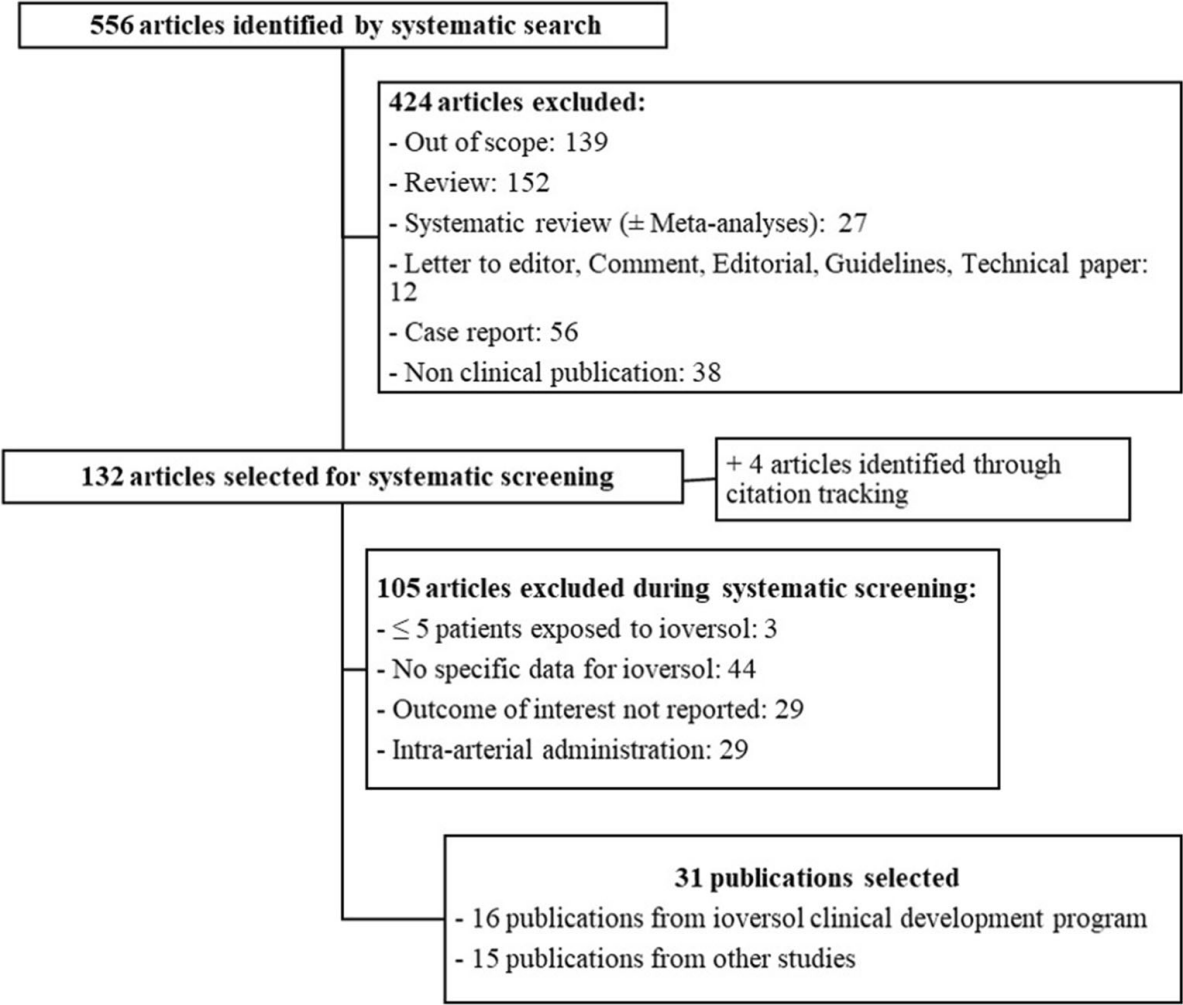
Flow diagram of the search strategy and study selection

Among the 556 articles identified, 132 underwent a full-text screening and 4 articles were identified through citation tracking [16–19]. Finally, 31 articles were included: 16 related to the ioversol clinical development program [20–35] and 15 from other studies (Fig. 1). Twenty-five studies had a prospective design and 11 were RCT [20–25, 28, 29, 31, 36, 37]. Four studies were on pediatric patients [27, 30, 38, 39].

The NOS was applied to all non-RCT and one RCT (randomized for patient hydration and not for ICM allocation) [36], indicating high quality for 4 studies and medium quality for 18 studies. All RCTs had a low risk of bias, except one [37] where some concerns linked to a potential performance bias were raised as the study was not double blinded.

Twenty-nine studies indicated the number of patients exposed to ioversol (total of 57,837 patients, including 13,484 pediatric patients) while two studies indicated the number of administered doses of ioversol, with more than 1.5 million in An et al [17] and 20,958 doses in Morales et al [40] (Table 1).

**Table 1:** Description of all selected studies

Study	Country	Study Design	Indication & Population	Contrast Media	Dose	N patients	Outcome	Quality Score ^a^
Clinical development program of ioversol								
McClennan 1989 [41]	USA	DB, RCT, S	Adult patients who underwent body CT	Ioversol 320	100.4 (33.7) mL	40	ADRs	Low risk of bias
				Diatrizoate 370	84.9 (28.6) mL	40		
Chagnaud 1992 [31]	France	DB, RCT, S	Adult patients who underwent body CT	Ioversol 300	146 (12.8) mL	41	ADRs	Low risk of bias
				Iopamidol 300	145 (15.4) mL	39		
Kopecky 1989 [32]	USA	OL, S	Adult patients who underwent body CT	Ioversol 320	141 (75–150) mL	42	ADRs	5
Sartor 1989 [33]	USA	OL, S	Adult patients who underwent head CT	Ioversol 320	132.4 mL	60	ADRs	5
Gillard 1992 [34]	France	OL, S	Adult patients who underwent head CT	Ioversol 300	60 mL	92	ADRs	5
Théron 1991 [35]	France	OL, S	Adult patients who underwent head CT	Ioversol 350	1 (0.1) mL/kg	40	ADRs	5
Wilkins 1990 [20]	England	DB, RCT, S	Adult patients who underwent venography	Ioversol 240	84.8 (40–200) mL	25	ADRs	Low risk of bias
				Iohexol 240	88.4 (40–150) mL	25		
Wilson 1989 [22]	USA	DB, RCT, S	Adult patients who underwent venography	Ioversol 240	NR	25	ADRs	Low risk of bias
				Iothalamate 202		25		
Scott 1990 [23]	Australia	RCT, S	Adult patients who underwent venography	Ioversol 240	71.3 (35–160) mL	26	ADRs	Low risk of bias
				Ioversol 320	76.3 (35–140) mL	26		
Colthurst 1990 [24]	England	DB, RCT, S	Adult patients who underwent urography	Ioversol 320	76.2 mL	40	ADRs	Low risk of bias
				Iohexol 300	76.6 mL	40		
Voegeli 1992 [25]	Switzerland	DB, RCT, S	Adult patients who underwent urography	Ioversol 350	50 mL	42	ADRs	Low risk of bias
				Iohexol 350	50 mL	35		
Lemaitre 1992 [26]	France	OL, S	Adult patients who underwent urography	Ioversol 350	45-100 mL	100	ADRs	5
Rieser 1992 [29]	Germany	DB, RCT, S	Adult patients who underwent intravenous DSA	Ioversol 300	176.2 mL	41	ADRs	Low risk of bias
				Iohexol 300	182.2 mL	39		
Wilkins 1989 [21]	England	SB, RCT, S	Healthy volunteers undergoing pharmacokinetic study	Ioversol 320	50–100–150 mL	18	ADRs	7
				Saline		6		
Montagne 1992 [27]	France	OL, S	Pediatric patients who underwent urography	Ioversol 300	2 (1–3) mL/kg	25	ADRs	5
Panuel 1992 [30]	France	OL, S	Pediatric patients who underwent body CT	Ioversol 300	2.8 (0.1) mL/kg	40	ADRs	5
Other studies								
Vogl 2012 [42]	Germany	O, P, M	Adult patients who underwent contrast-enhanced CT	Ioversol 160, 240, 300, 320, 350	NR	10,836	ADRs Anaphylactoid reactions	5
An 2019 [17]	Korea	R, M	Adult patients who underwent contrast-enhanced CT	Ioversol	NR	1,592,523 ^**b**^	ADRs	6
				Iohexol		3,816,072		
				Iopamidol		2,333,794		
				Iopromide		1,310,393		
				Iomeprol		1,042,096		
				Iobitridol		938,251		
				Iodixanol		679,667		
Chen 2017 [43]	China	R, S	Adult patients who underwent contrast-enhanced CT	Ioversol (Optiray)	NR	5261	ADRs	6
				Ioversol (Hengrui)		105		
				Iohexol (Omnipaque)		12,824		
				Iohexol (Ousu)		18,773		
				Iopamidol		18,044		
				Iopromide		17,616		
				Iodixanol		5219		
Morales 2017 [40]	Spain	P, S	Adult patients ^c, d^	Ioversol	NR	20,958 ^b^	HSRs	5
				Iopamidol		54,453		
				Iomeprol		17,645		
Cha 2019 [41]	Korea	P, M	Adult patients who underwent contrast-enhanced CT	Ioversol 240, 320, 350	NR	24,220	HSRs	6
				Iopromide 370		7335		
				Iopamidol 300, 370		53,037		
				Iomeprol 350, 400		29,247		
				Iohexol 240, 300, 350		51,586		
				Iodixanol 270, 320		3043		
				Iobitrodol 300, 350		27,613		
Gomi 2010 [37]	Japan	P, RCT, S	Adult patients who underwent contrast-enhanced CT	Ioversol 320	2 mL/kg	1886	Acute ADRs	Some concerns
				Iomeprol 300		1751		
				Iopamidol 300		1697		
				Iohexol 300		1792		
				Iopromide 300		1805		
Juchem 2007 [18]	Brazil	P, S	Adult patients who underwent contrast-enhanced CT	Ioversol	NR	190	Acute ADRs	6
				Meglumine diatrizoate		161		
Motosugi 2016 [36]	Japan	P, RCT ^e^, S	Adult patients who underwent contrast-enhanced abdominal and pelvic CT	Ioversol 320	No reaction 583.8 ± 44.7 mgI/Kg	440	Acute allergic-like and physiologic reactions	6
				Iohexol 300		1722		
				Iopamidol 370	Reaction 576.8 ± 42.4 mgI/Kg	1298		
				Iomeprol 350		1028		
Federle 1998 [43]	USA	P, S	Adult patients who underwent contrast-enhanced thorax or abdomen CT	Slow injection rate	150 mL in 92% of cases		Anaphylactoid reactions	7
				Ioversol 320		250		
				Iothalamate		725		
				Fast injection rate				
				Ioversol 320		202		
				Iothalamate		650		
Callahan 2009 [38]	USA	R, S	Pediatric and young adults (up to 21 years old) who underwent contrast-enhanced CT or excretory urography.	Ioversol 320	1.5–2 mL/Kg	12,494	ADRs	5
Louvel 1996 [46]	France	P, S	Contrast-enhanced CT in geriatric population	Ioversol 300	Age > 69 years old 1.36 ± 0.06 mL/Kg	47	PC-AKI	5
					Age < 60 years old 1.39 ± 0.08 mL/Kg	44		
Ng 2010 [47]	USA	R, S	Head and torso CT in oncologic patients	Ioversol 320	95-150 mL	81	PC-AKI	8
				Unenhanced CT		81		
Gomez 2013 [19]	Spain	P, S	Contrast-enhanced CT in diabetic patients	Ioversol 320	Mean: 100 mL Maximum: 150 mL	98	PC-AKI	5
Moura 2017 [16]	Brazil	R, S	Patients undergoing examination with IV contrast injection, with a length of stay in ICU > 3 days	Ioversol 320	92.9 ± 10.3 mL	140	PC-AKI	5
Gilligan 2020 [39]	USA	R, S	Hospitalized pediatric patients undergoing contrast-enhanced CT or abdominal US	Ioversol 320	1.5–2 mL/kg	925	PC-AKI	7
				Unenhanced US		925		

*ADRs* Adverse drug reactions; *HSRs* Hypersensitivity reactions; *PC-AKI* Post-contrast acute kidney injury; *P* Prospective; *R* Retrospective; *RCT* Randomized Controlled Trial; *S* single-center; *M* Multicenter; *ICU* Intensive care unit; *eGFR* estimated Glomerular Filtration Rate; *IV* Intravenous; *NR* Not reported

^a^Quality score according to Newcastle-Ottawa Scale (NOS) or revised Cochrane Risk of Bias assessment tool for randomized trials (ROB 2) algorithm

^b^Number of administered doses of contrast media

^c^No specification of route of administration in the publication

^d^Age and gender reported only for 329 patients who experienced HSRs

^e^Randomization for hydration but not for contrast allocation

In adult studies conducted during the clinical development of ioversol, the mean administered dose ranged between 50 and 176 mL, while sparse information was retrieved from the other adult studies. In pediatric patients, the injected dose was 1–3 mL/kg [27, 30, 38, 39].

Among the selected studies, 26 [17, 18, 20–38, 40, 42–45] documented the incidence of all ADRs or specifically HSRs (56,502 patients and 1,613,481 doses) and 5 studies [16, 19, 39, 46, 47] reported the incidence of PC-AKI (1335 patients). Contrast-enhanced CT was the main indication for which ioversol was used, followed by venography and urography. The mean age was 28–78 years old in adult studies and 5–10 years old in pediatric studies.

Twelve publications reported information on intensity of reactions (Table 2), with detailed information on the methodology of classification in 4 of them (Table 3). In addition, 4 publications reported information on seriousness of reactions (Table 2).

**Table 2:** Incidence of ADRs/HSRs after intravenous administration of ioversol

Study	Contrast Media	*N* Patients	Type of Reaction	Incidence (%)	Incidence of Serious/Severe Reactions (%)
McClennan 1989 [41]	Ioversol	40	ADRs	0%	None severe
	Diatrizoate	40		35%	
Chagnaud 1992 [31]	Ioversol	41	ADRs	63.4%^i^	None severe
	Iopamidol	39		69.2%^i^	
Kopecky 1989 [32]	Ioversol	42	ADRs	0%	-
Sartor 1989 [33]	Ioversol	60	ADRs	3.3%	None severe
Gillard 1992 [34]	Ioversol	92	ADRs	42.4%^i^	None severe
Théron 1991 [35]	Ioversol	40	ADRs	12.5%	None severe
Wilkins 1990 [20]					Severe ADRs
	Ioversol	25	ADRs	4%	4%
	Iohexol	25		0%	0%
Wilson 1989 [22]	Ioversol	25	ADRs	0%	None serious
	Iothalamate	25		4%	
Scott 1990 [23]	Ioversol	26	ADRs	0%	-
	Ioversol	26			
Colthurst 1990 [24]	Ioversol	40	ADRs	0%	None serious
	Iohexol	40		2.5%	
Voegeli 1992 [25]	Ioversol	42	ADRs	0%	-
	Iohexol	35		0%	
Lemaitre 1992 [26]	Ioversol	100	ADRs	1^st^ injection 12%^h^ 2^nd^ injection 6.3%^h^	NR
Rieser 1992 [29]	Ioversol	41	ADRs	4.9%	NR
	Iohexol	39		5.1%	
Wilkins 1989 [21]			ADRs		Severe ADRs
	Ioversol	18		33.3%	0%
	Saline	6		16.7%	16.7%
Montagne 1992 [27]	Ioversol	25	ADRs	4%	NR
Panuel 1992 [30]	Ioversol	40	ADRs	5%	NR
Vogl 2012 [42]	Ioversol	10836	ADRs	0.28%	Serious ADRs 0.037%
			Anaphylactoid reactions	0.18%	Serious anaphylactoid reactions 0.028%
An 2019 [17]			ADRs		Serious ADRs ^**c**^
	Ioversol	1592523 ^b^		0.23%	0.01%
	Iohexol	3816072		0.24%	0.01%
	Iopamidol	2333794		0.30%	0.02%
	Iopromide	1310393		0.59%	0.03%
	Iomeprol	1042096		0.70%	0.05%
	Iobitridol	938251		0.55%	0.02%
	Iodixanol	679667		0.27%	0.03%
Chen 2017 [43]			ADRs		Moderate/Severe ADRs ^d^
	Ioversol (Optiray)	5261		0.13%	0.02%
	Ioversol (Hengrui)	105		0.95%	0.00%
	Iohexol (Omnipaque)	12824		0.23%	0.02%
	Iohexol (Ousu)	18773		0.31%	0.04%
	Iopamidol	18044		0.25%	0.06%
	Iopromide	17616		0.61%	0.02%
	Iodixanol	5219		0.67%	0.48%
Morales 2017 [40] ^a^	Ioversol	20958 ^b^	HSRs	0.2%	NR
	Iopamidol	54453		0.14%	
	Iomeprol	17645		0.4%	
Cha 2019 [44]			HSRs		Severe HSR ^e^
	Ioversol	24220		0.66%	0.00%
	Iopromide	7335		0.37%	0.00%
	Iopamidol	53037		0.70%	0.01%
	Iomeprol	29247		0.95%	0.01%
	Iohexol	51586		0.62%	0.01%
	Iodixanol	3043		0.99%	0.07%
	Iobitrodol	27613		0.89%	0.01%
Gomi 2010 [37]	Ioversol	1886	Acute ADRs	1.80%	NR
	Iomeprol	1751		3.90%	
	Iopamidol	1697		2.20%	
	Iohexol	1792		2.00%	
	Iopromide	1805		3.50%	
Juchem 2007 [18]	Ioversol	190	Acute ADRs	1.0%^c,f^	None severe
	Meglumine diatrizoate	161		12.4% ^g^	
Motosugi 2016 [36]	Ioversol	440	Acute allergic-like reactions	1.8%	None severe ^e^
				2.0%	
	Iohexol	1722		2.0%	
				3.6%	
	Iopamidol	1298	Acute physiologic reactions	1.1%	
				1.6%	
	Iomeprol	1028		2.5%	
				2.7%	
Federle 1998 [45]	Slow injection rate		Anaphylactoid reactions		NR
	Ioversol	250		2.0% ^c^	
	Iothalamate	725		8.3%	
	Fast injection rate				
	Ioversol	202		2.5% ^c^	
	Iothalamate	650		9.1%	
Callahan 2009 [38]	Ioversol	12494	ADRs	0.46%	None severe ^e^

*NR* Not reported; *ADRs* Adverse drug reactions; *HSRs* Hypersensitivity reactions

^a^No specification of route of administration in the publication

^b^Number of administered doses of contrast media

^c^Statistically significant difference

^d^According to guidelines for iodinated contrast agents use of Chinese Society of Radiology

^e^According to American College of Radiology Manual on Contrast Media

^f^Only 2 cases of vomiting

^g^85% of the reactions were anaphylactoid

^h^Excluding heat sensation

^i^Including heat sensation

**Table 3:** Event classification by intensity

Study	Outcome	Main source of classification	Mild	Moderate	Severe
Callahan 2009 [38]	ADRs	ACR Manual on Contrast Media (5^th^ edition)	Itching, hives or rash, flushing, nasal congestion	Tachycardia, bradycardia, hypertension, hypotension, pronounced cutaneous reaction, dyspnea, wheezing	Laryngeal edema, cardiopulmonary arrest, profound hypotension, unstable arrhythmias, convulsions, unresponsiveness
Chen 2017 [43]	ADRs	CSR guidelines for iodinated contrast agents use	Cough, sneezing, nasal congestion, transient chest tightness, conjunctivitis, rhinitis, nausea, systematic fever, urticaria, itching, angioneurotic edema, mild or localized facial swelling, mild trembling or shivering, single symptom such as mild gastrointestinal discomfort, feeling of binaural blockage, transient blurred vision, dizziness, and numb limbs	Severe vomiting, systematic urticaria, moderate or substantial facial swelling, dyspnea, and vasovagal reaction, single systematic trembling or shivering, hypertension, chest distress, palpitation	Laryngeal edema, seizure, trembling, convulsions, single trembling or shivering coupled with severe systematic symptoms, oxygen desaturation unconsciousness, shock, death
Morales 2017 [40]	HSRs	Brown grading [48]	Generalized erythema, urticaria, periorbital edema, angioedema	Dyspnea, stridor, wheeze, nausea, vomiting, dizziness (presyncope), diaphoresis, chest or throat tightness, abdominal pain	Cyanosis or SpO_2_ ≤ 92%, hypotension, confusion, collapse, loss of consciousness, or incontinence
Cha 2019 [44]	HSRs	ACR Manual on Contrast Media (10^th^ edition)	Limited urticaria and pruritis, limited cutaneous edema, itching or scratchy throat, nasal congestion, sneezing, conjunctivitis, rhinorrhea	Diffuse urticaria and pruritis, diffuse erythema with stable vital signs, facial edema without dyspnea, throat tightness or hoarseness without dyspnea, wheezing or bronchospasm with mild or no hypoxia	Diffuse edema or facial edema with dyspnea, diffuse erythema with hypotension, anaphylactic shock with hypotension and tachycardia, wheezing or bronchospasm with marked hypoxia

*ADRs* adverse drug reactions, *HSRs* hypersensitivity reactions, *CSR* Chinese Society of Radiology, *ACR* American College of Radiology

### Adverse drug reactions and hypersensitivity reactions

The overall incidence of ADRs in adults was reported in 15 studies [17, 20–26, 28, 29, 32, 33, 35, 42, 43] with a median of 0.23%. In two studies where heat sensation was assessed in a specific questionnaire, a higher incidence of ADRs was reported (42–63%) [31, 34].

In 12 studies of ioversol clinical development (658 patients), the median incidence of ADRs was 1.65% (range: 0–33.3%), with 6 studies reporting no ADRs (Table 2). The highest incidence was reported in a pharmacokinetic study [21], where 6 of 18 patients reported ADRs, none of which was severe. Overall, most of the reported ADRs were minor and consisted of nausea, vomiting, and headache.

Three other studies reported incidences between 0.13 and 0.23% [17, 42, 43]. Vogl et al [42] reported ADRs in 0.28% of 10,836 patients, mainly urticaria (0.12%), nausea (0.10%), and erythema (0.06%). Four serious ADRs (0.037%) were reported, including 3 anaphylactoid reactions requiring hospitalization (0.028%).

An et al [17] reported an incidence of ADRs with ioversol of 0.23%, with urticaria (47.3%) and itching (43.9%) being the most frequent acute ADRs, and maculopapular rash (88%) the most frequent delayed ADR. The incidence of serious ADRs with ioversol was 0.01% (no deaths reported) (Table 2).

Chen et al [43] showed that ADRs were mainly evocative of HSRs, with an incidence of 0.13% for ioversol. Only one anaphylactic shock reaction (0.019%) and no case of laryngeal edema was reported with ioversol for 5261 patients exposed. The incidence of moderate and severe ADRs with ioversol was 0.02%, no deaths induced by ICM were reported, and all ADRs resolved.

The incidence of HSRs with ioversol was explicitly reported in two studies (0.2–0.66%) [40, 44] (Table 2). Morales et al [40] included patients with a previous history of HSRs to ICM. The incidence of HSRs was 0.2% with ioversol (mostly cutaneous symptoms [88.7%]), and severe HSRs represented 6.4% of all cases (no specific data with ioversol). In the study by Cha et al [44], HSR incidence was 0.66% and no severe HSRs were reported among 24,220 patients who received ioversol.

The incidence of acute ADRs was explicitly reported in two studies [18, 37], and in a third study, acute ADRs represented the majority of the reported ADRs (88.6%) [17]. The incidence was 0.23–1.8% [17, 18, 37]. In the study by Gomi et al [37], the acute ADR incidence was significantly lower with ioversol (1.8%) compared to iomeprol (3.9%) and iopromide (3.5%). Overall, 0.7% of the reported reactions required treatment and resolved, with no association with the type of ICM. No patient experienced life-threatening severe complications requiring immediate transfer to the emergency department.

In the study by Juchem et al [18], acute ADRs corresponding to two cases of vomiting (1%) were reported with ioversol, while the incidence of acute ADRs with meglumine diatrizoate was 12.5% (85% were anaphylactoid reactions). All acute ADRs were mild and patients recovered spontaneously.

Furthermore, in the study by Motosugi et al [36], acute allergic-like reaction incidence with ioversol was 1.8% and that of acute physiologic reactions was 1.1%, and none were severe.

Anaphylactoid reaction incidence in patients exposed to ioversol was reported in two studies ranging from 0.18% [42] to 2.5% [45]. Federle et al [45] reported more than a threefold higher incidence of anaphylactoid reactions with iothalamate compared to ioversol at both slow (8.3% vs. 2.0%, respectively) and fast (9.1% vs. 2.5%, respectively) injection rates.

The incidence of ADRs in pediatric patients exposed to ioversol for CT or urography was reported by Callahan et al [38], with a total of 12,494 pediatric patients and a mean (SD) age of 9.5 (5.9) years. Mild symptoms such as nausea, warm sensation, altered taste, and anxiety were not recorded as ADRs in this study. No ADRs were reported among 941 patients who underwent excretory urography. Only mild (0.38%) and moderate ADRs (0.08%) were reported. In patients aged ≤ 6 years old, only ADRs of mild intensity were reported. Two other pediatric studies from ioversol clinical development (mean age ≈ 5 years) reported ADRs in 3 of 65 patients (4.6%): metallic taste, nausea, and vomiting in two patients and not defined in the third patient [27, 30].

## Studies with a comparison with other ICM

Ioversol was compared to a non-ionic, monomeric LOCM in 5 studies [20, 24, 25, 29, 31] during its clinical development, and no difference was shown regarding ADR incidence (Table 2). In 6 other studies [17, 36, 37, 40, 43, 44], the incidence of all ADRs and HSRs and severe/serious events (when reported) with ioversol was among the lowest (Table 2). In 3 studies [17, 43, 44], also including data with the IOCM iodixanol, the incidences of ADRs and HSRs with ioversol were 0.13–0.66% vs. 0.27–0.99% with iodixanol, and severe/serious events were 0.00–0.02% vs. 0.03–0.48%, respectively.

Five studies reported that the incidence of ADRs or HSRs was significantly different between ICM, with the highest incidences reported with iomeprol and/or iopromide [17, 37, 40, 43, 44]. Two studies compared the nature of ADRs between ICM. In Chen et al, rash was the predominant ADR reported with all ICM, but was more frequent with iodixanol. Facial swelling was more often reported with iodixanol compared with iopamidol and iopromide and was not reported with ioversol [43]. An et al analyzed the prevalence of ADRs by system organ class (SOC) and reported that “skin and appendages disorders” were more frequent with iodixanol, and “gastrointestinal system disorders” and “respiratory system disorders” more frequent with iomeprol [17].

### Post-contrast acute kidney injury

PC-AKI prophylactic measures were described in two studies, and consisted of oral or IV hydration [16, 19]. A large heterogeneity in PC-AKI incidence was observed among the 5 studies (1–42%), due to heterogenous patient populations and differences in used PC-AKI definitions (Table 4).

**Table 4:** Incidence of PC-AKI after intravenous administration of ioversol

Study	Contrast Media	N Patients	PC-AKI Definition	Incidence (%)
Louvel 1996 [46]	Ioversol	Total: 91	sCr rise > 25% within 72 hours	1.1%
		Age > 69 years old: 47		2.1%
		Age < 60 years old: 44		0%
Ng 2010 [47]	Ioversol	81	sCr rise > 0.3 mg/dL or > 50% within 7 days	17%
	Unenhanced CT	81		17%
Gomez 2013 [19]	Ioversol	98	sCr rise > 0.5 mg/dL	1%
Moura 2017 [16]	Ioversol	140	sCr rise ≥ 0.5 mg/dL or > 25% within 72 hours	12.1%
			sCr rise > 0.3 mg/dL or > 50% within 48 hours	42.1%
			KDIGO stage 1 (×1.5 sCr rise)	23.5%
			KDIGO stage 2 (×2 sCr rise)	8.5%
			KDIGO stage 3 (×3 sCr rise)	12.1%
Gilligan 2020 [39]	Ioversol	925	sCr rise ≥ 0.3 mg/dL or ≥ 50% within 48h	2.4%
	Unenhanced US	925		2.6%

*KDIGO* Kidney Disease Improving Global Outcomes; *ICU* intensive care unit; *sCr* Serum creatinine

In Louvel et al [46], one patient (1.1%) aged 82 years had a 25% increase in sCr (87 to 109 mmol/L) which rapidly improved. An increase > 10% in sCr was observed in 8 patients aged > 69 years and 4 patients aged < 60 years, with no significant difference between the two age groups. In Gomez et al [19] (98 diabetic patients using metformin), PC-AKI was observed for only one patient (1%) with an eGFR < 60 mL/min/1.73 m^2^ (incidence of 4.7% in this subpopulation), without clinical repercussion. During a 1-month follow-up period, no patient had alteration of renal function requiring medical care.

Ng et al [47] included two matched groups of patients who underwent CT with or without ioversol, and showed no difference in PC-AKI incidence (17%), sCr increase (0.25 and 0.11 mg/dL, respectively), need for hemodialysis (2% and 1%, respectively), and in-hospital mortality (17% and 21%, respectively). Moura et al [16] included a high-risk population of patients admitted to intensive care unit (ICU) with a length of stay > 3 days. The broader PC-AKI definition used in this study resulted in an incidence of 42%. Hemodialysis was needed for seven patients (12%) and deaths reported for 9 patients (6.5%).

Gilligan et al [39] included two matched groups of pediatric patients exposed to ioversol (aged 8 [6] years), and those who underwent abdominal US, and showed no difference in PC-AKI incidence (2.4% and 2.6%, respectively). In patients with an eGFR < 60 mL/min/1.73 m^2^, PC-AKI incidence was lower with ioversol (5.6% vs. 11.1%, respectively), although not statistically significant.

## Discussion

This systematic literature review showed a large heterogeneity between studies regarding the way ADRs were collected and the type of ADRs reported. The median (range) incidence of ADRs with IV ioversol was 0.23% (0–33.3%). This variability is mainly emanating from ioversol clinical development studies, which included a low number of patients, and where heat and pain were specifically assessed in some studies. In the other studies, the incidence of ADRs in adults was low, independent of the type of ADR reported: 0.13–0.28% for all ADRs [17, 42, 43], 0.23–1.8% for acute ADRs [17, 18, 36, 37], and 0.2–0.66% for HSRs [40, 44]. In two studies, the relatively high incidence of events could be due to the systematic interview of patients [36] and a higher incidence of mild events (> 90% [36], 83% [44]). These incidences are comparable to those reported with other ICM. Indeed, two large retrospective studies with more than 246,000 patients who received IV non-ionic LOCM, reported an ADR incidence of 0.3% [49, 50].

The incidence of severe reactions to IV ioversol was low (0–0.02%) [18, 36, 38, 43, 44] and similar (if not lower) to what has been reported with other ICM (0.01–0.08%) [49–52]. Anaphylactic shock was reported in only one study, with a low incidence (0.019%) [43], consistent with a previous study using other non-ionic ICM (0.016%) [53]. Thus, the occurrence of severe events can be considered as rare with non-ionic ICM.

The risk of ADRs after using ICM in pediatric patients, and particularly life-threatening reactions, is low [54, 55]. Callahan et al reported a low incidence of ADRs (0.46%) and absence of severe events [38]. In one study, where non-ionic ICM were administered in 13,461 pediatric patients, the overall incidence of ADRs was 3.4%, and that of severe ADRs was 0.07% [55]. Another study reported an incidence of allergic-like reactions of 0.18% overall and 0.027% for severe reactions on 11,306 IV administrations [56]. This variability could be due to the different reporting (all ADRs or specific types, some mild symptoms not recorded as ADRs) [38]. ADR incidence was previously associated with the age of the patients with lower incidences observed in patients aged ≤ 10 years (0.22%) [50]. This could be linked to weak immune responses in pediatric patients compared to adults. Overall, it can be concluded that ioversol has a similar safety profile as other non-ionic ICM when IV administered to pediatric patients.

Several large retrospective studies investigated the safety profile of different ICM. Two studies using different non-ionic ICM reported that cutaneous and gastrointestinal disorders were the most frequent for mild events (51–69% and 12–14%, respectively) [49, 50]. In contrast, in a comparison of the safety profile of seven ICM, it was reported that skin (69.4%) and respiratory system disorders (8.9%) were the most frequent, followed by gastrointestinal disorders (5.7%). For ioversol, the proportion of gastrointestinal disorders and cardiovascular disorders was significantly higher than the general profile of LOCM (8% vs. 6% and 2% vs. 1%, respectively) and skin disorders significantly lower (65% vs. 70%) [57]. Despite some differences between LOCM, cutaneous and gastrointestinal manifestations are the most frequent and it could be concluded that ioversol has a similar safety profile to other LOCM.

PC-AKI incidence was highly variable, with the highest incidence reported in a critical care population with strong competing risk factors for AKI [16]. It is advised to use the lowest dose of ICM as possible in patients with diabetes and other co-morbidities and/or in patients with impaired renal function [7, 58, 59]. Consistent with what has been reported by Gomez et al [19], others reported a PC-AKI incidence of 1% in patients with normal renal function, which increased to 14% in those with severe renal impairment [60].

In the two studies comparing CT with ioversol to unenhanced CT or abdominal US, IV administration of ioversol *per se* did not increase the incidence of PC-AKI in adult and pediatric patients [39, 47]. Others reported that IV ICM administration for CT was not associated with an increased risk of PC-AKI [60], and large retrospective studies using propensity score matching suggested a lower incidence of PC-AKI than previously estimated [61]. In studies comparing the safety profile of iodixanol to that of other non-ionic LOCM, urinary system disorders were more frequently reported than with non-ionic LOCM [57]. However, this could be due to iodixanol being used more frequently in high-risk patients with underlying renal diseases [17]. The proportion of urinary system disorders with ioversol was comparable to the general profile of LOCM, suggesting a similar safety profile with regard to PC-AKI [17, 57]. In procedures involving IV administration of ICM, several meta-analyses showed that iodixanol was not associated with a reduction in PC-AKI compared to non-ionic LOCM [62–64].

In conclusion, the safety profile of ioversol, by IV route, is good and comparable to that of other non-ionic LOCM, with a low incidence of ADRs overall and particularly severe/serious ADRs, in adult and pediatric patients. PC-AKI incidence following IV administration of ioversol was not higher than in patients unexposed to ICM. Further well-designed studies are warranted in order to confirm these results.

## Supplementary Information

ESM 1 (DOCX 20 kb)

## Abbreviations

ADR: Adverse drug reaction
AKIN: Acute kidney injury network
CIN: Contrast-induced nephropathy
CKD: Chronic kidney disease
eGFR: Estimated glomerular filtration rate
ESUR: European Society of Urogenital Radiology
HOCM: Hyperosmolar contrast medium
HSR: Hypersensitivity reaction
IA: Intra-arterial
ICM: Iodinated contrast media
ICU: Intensive care unit
IOCM: Iso-osmolar contrast medium
IV: Intravenous
KDIGO: Kidney Disease Improving Global Outcomes
LOCM: Low-osmolar contrast medium
NOS: Newcastle-Ottawa Scale
PC-AKI: Post-contrast acute kidney injury
PRISMA: Preferred Reporting Items for Systematic Reviews and Meta-analyses
RCT: Randomized controlled trial
ROB 2: The revised Cochrane risk of bias assessment tool
sCr: Serum creatinine
SOC: System organ class

## Declarations

### Guarantor

The scientific guarantor of this publication is Aart J. van der Molen.

### Statistics and Biometry

No complex statistical methods were necessary for this paper.

### Study subjects or cohorts overlap

Studies with duplicate data were excluded from this systematic review.

### Methodology

• Multicenter study
