# Supplementary material for: A systematic review of the incidence of hypersensitivity reactions and post-contrast acute kidney injury after ioversol in more than 57,000 patients: part 1—intravenous administration
Source: Eur Radiol. 2022 Mar 21;32(8):5532–45. doi: 10.1007/s00330-022-08636-3 (PMC9279198; doi:10.1007/s00330-022-08636-3)
Supplement: Supplementary file 1 — (DOCX 20 kb) [file 330_2022_8636_MOESM1_ESM.docx]

**Pubmed :**

((ioversol OR optiray OR optiject OR isoversol OR ultraject) AND (("contrast media/adverse effects"[MeSH Terms] OR "contrast media/poisoning"[MeSH Terms] OR "contrast media/toxicity"[MeSH Terms] OR "pregnancy"[Title/Abstract] OR "pregnanc*"[Title/Abstract] OR "pregnant"[Title/Abstract] OR "fetus"[Title/Abstract] OR "fetal"[Title/Abstract] OR "embryo"[Title/Abstract] OR "prenatal"[Title/Abstract] OR "reproduction"[Title/Abstract] OR "fertilit*"[Title/Abstract] OR "abortion"[Title/Abstract] OR "infertilit*"[Title/Abstract] OR "placenta"[Title/Abstract] OR "placental"[Title/Abstract] OR "transplacental"[Title/Abstract] OR "lactation"[Title/Abstract] OR "breast feeding"[Title/Abstract] OR "breast milk"[Title/Abstract] OR "pharmacovigilance"[Title/Abstract] OR "postmarketing surveillance"[Title/Abstract] OR "safety"[Title/Abstract] OR "toxicit*"[Title/Abstract] OR "tolerance"[Title/Abstract] OR "tolerability"[Title/Abstract] OR "toxic"[Title/Abstract] OR "death"[Title/Abstract] OR "mortal"[Title/Abstract] OR "mortalit*"[Title/Abstract] OR "lethal"[Title/Abstract] OR "lethalit*"[Title/Abstract] OR "fatal"[Title/Abstract] OR "fatalit*"[Title/Abstract] OR "morbidit*"[Title/Abstract] OR "neurotoxicit*"[Title/Abstract] OR "immunotoxicit*"[Title/Abstract] OR "cardiotoxicit*"[Title/Abstract] OR "cytotoxicit*"[Title/Abstract] OR "immunocytotoxicit*"[Title/Abstract] OR "hepatotoxicit*"[Title/Abstract] OR "carcinogenic*"[Title/Abstract] OR "cancerogenic*"[Title/Abstract] OR "teratogenic*"[Title/Abstract] OR "mutagenic*"[Title/Abstract] OR ((("adverse"[Title/Abstract] OR "side"[Title/Abstract] OR "secondary"[Title/Abstract] OR "undesirable"[Title/Abstract] OR "unwanted"[Title/Abstract]) AND ("effect"[Title/Abstract] OR "event"[Title/Abstract] OR "reaction"[Title/Abstract] OR "effects"[Title/Abstract] OR "events"[Title/Abstract] OR "reactions"[Title/Abstract])) OR "allergy"[Title/Abstract] OR "allergic"[Title/Abstract] OR "anaphylaxis"[Title/Abstract] OR "anaphylactic"[Title/Abstract] OR "anaphylactoid"[Title/Abstract] OR "hypersensitivity"[Title/Abstract] OR "extravasated"[Title/Abstract] OR "extravasation"[Title/Abstract] OR "extravasations"[Title/Abstract] OR "intravasated"[Title/Abstract] OR "intravasation"[Title/Abstract] OR "intravasations"[Title/Abstract] OR "poisoning"[Title/Abstract] OR "unexpected therapeutic"[Title/Abstract] OR "environmental exposure"[Title/Abstract] OR "environmental safety"[Title/Abstract] OR "contraindication"[Title/Abstract] OR "contraindications"[Title/Abstract] OR "chemically induced"[Title/Abstract] OR "contrast medium induced"[Title/Abstract] OR "contrast media induced"[Title/Abstract] OR "contrast induced"[Title/Abstract] OR "encephalopathy"[Title/Abstract] OR "encephalopathies"[Title/Abstract] OR "lack of drug effect"[Title/Abstract] OR "patient exposure"[Title/Abstract] OR "class effect"[Title/Abstract] OR "class effects"[Title/Abstract] OR "medication error"[Title/Abstract] OR "medication errors"[Title/Abstract] OR "misuse"[Title/Abstract] OR "misuses"[Title/Abstract] OR "lack of efficacy"[Title/Abstract] OR "overdose"[Title/Abstract] OR "overdosage"[Title/Abstract] OR "inadvertent"[Title/Abstract] OR "accidental"[Title/Abstract] OR "wrong administration"[Title/Abstract] OR "drug interaction"[Title/Abstract] OR "drug interactions"[Title/Abstract] OR "off label"[Title/Abstract] OR "nephropathy"[Title] OR "nephrotoxicit*"[Title] OR "kidney injury"[Title] OR "kidney injuries"[Title] OR "renal injury"[Title] OR "renal injuries"[Title] OR "occupational exposure"[Title/Abstract] OR "occupational safety"[Title/Abstract] OR "infectious"[Title/Abstract] OR "nosocomial"[Title/Abstract])))) AND (human OR man OR men OR woman OR women OR boy OR girl OR child* OR patient*)

**EMBASE :**

('adverse drug reaction'/exp OR 'adverse event'/exp OR 'drug toxicity and intoxication'/exp OR 'side effect'/exp OR 'pharmacovigilance'/exp OR 'postmarketing surveillance'/exp OR 'drug interaction'/exp OR 'drug safety'/exp OR 'allergic reaction'/exp OR 'drug eruption'/exp OR 'drug hypersensitivity'/exp OR 'chemically induced disorder'/exp OR 'drug contraindication'/exp OR 'pregnancy'/exp OR 'pregnancy complication'/exp OR 'pregnancy disorder'/exp OR 'pregnancy rate'/exp OR 'fetus'/exp OR 'fetus development'/exp OR 'embryo development'/exp OR 'prenatal':ab,ti OR 'placental transfer'/exp OR 'lactation'/exp OR 'breast milk'/exp OR 'breast feeding'/exp OR 'drug milk level'/exp OR 'reproduction'/exp OR 'abortion'/de OR 'fertility'/de OR 'infertility'/de OR 'teratogenic agent'/exp OR 'medication error'/exp OR 'off label drug use'/exp OR 'drug overdose'/exp OR 'drug misuse'/exp OR 'drug retention'/exp OR 'contrast medium extravasation'/exp OR 'contrast induced nephropathy'/exp OR nephrotoxicity OR 'disease transmission'/exp OR 'drug tolerance'/exp OR 'unexpected therapeutic effect'/exp OR 'lack of drug effect'/exp OR 'environmental exposure'/exp OR 'occupational exposure'/exp OR 'occupational safety'/exp OR 'neurotoxicity'/exp OR 'immunotoxicity'/exp OR 'liver toxicity'/exp OR 'cardiotoxicity'/exp OR 'cytotoxicity'/exp OR 'immunocytotoxicity'/exp OR (patient NEAR/1 exposure) OR (class NEAR/1 effect*) OR (lack NEAR/1 efficacy) OR (off NEAR/1 label) OR (wrong NEAR/3 administration) OR (((side OR undesirable OR secondary OR unwanted) NEXT/2 (effect* OR reaction* OR event* OR outcome*)):ab,ti) OR safety:ti OR toxicit*:ti OR toxic:ti OR 'pharmacotoxicit*':ti OR 'hepatotoxicit*':ti OR death:ti OR mortal:ti OR mortality:ti OR fatal:ti OR fatality:ti OR lethal:ti OR lethality:ti OR morbidity:ti OR 'carcinogen*':ab,ti OR 'mutagen*':ab,ti OR 'cancerogen*':ab,ti OR 'organ dysfunction':ab,ti OR 'organ failure':ab,ti OR poisoning:ab,ti OR inadvertent:ab,ti OR accidental:ab,ti OR transmission:ab,ti OR nosocomial:ab,ti OR allergy:ab,ti OR allergic:ab,ti OR anaphylactic:ab,ti OR anaphylaxis:ab,ti OR anaphylactoid:ab,ti OR intravasated:ab,ti OR intravasation:ab,ti OR intravasations:ab,ti OR 'contrast media induced':ab,ti OR 'contrast medium induced':ab,ti OR 'contrast induced':ab,ti OR encephalopathy:ab,ti OR ((renal:ab,ti OR kidney:ab,ti) AND (injury:ab,ti OR injuries:ab,ti))) AND ('ioversol'/exp OR ioversol OR 'optiray'/exp OR optiray OR 'optiject'/exp OR optiject OR isoversol) AND [1989-2020]/py AND ('article'/it OR 'review'/it) AND ('human'/exp OR human OR m?n OR wom?n OR child OR boy OR girl)
